# Epithelioid Hemangioendothelioma with *WWTR1-CAMTA1* Fusion in the Parotid Gland Presenting as Bell's Palsy

**DOI:** 10.1155/2022/5687190

**Published:** 2022-06-24

**Authors:** Landon J. Kunzelman, Shweta Agarwal, Nathan Boyd, Cory J. Broehm

**Affiliations:** ^1^University of New Mexico, Department of Pathology, MSC08 4640, Reginald Heber Fitz Hall, Room 335, 1 University of New Mexico, Albuquerque, NM 87131, USA; ^2^University of New Mexico, Department of Surgery-Division of Otolaryngology-Head and Neck Surgery, MSC10 5610, 1 University of New Mexico, Albuquerque, NM 87131, USA

## Abstract

Epithelioid hemangioendothelioma is a rare tumor of endothelial differentiation most commonly arising in soft tissue, liver, and lung, following a variable clinical course. Most cases are characterized by a t(1;3)(p36;q23-25) resulting in *WWTR1-CAMTA1* fusion. Only five epithelioid hemangioendothelioma have been previously reported arising in the salivary glands. None have presented as Bell's palsy. In the current case, a 37-year-old female presented with a longstanding complaint of pain and fullness in the right preauricular region and progressive episodes of Bell's palsy and facial nerve weakness. Surgical resection showed a tumor comprised of atypical cells with occasional intracytoplasmic vacuoles in a fibromyxoid stroma. Immunohistochemical stains demonstrated the neoplastic cells expressed ERG, CD31, and CD34, confirming vascular differentiation. Fluorescence in situ hybridization revealed a t(1;3)(p36;q25), confirming a diagnosis of epithelioid hemangioendothelioma. At 12-month follow-up, the patient has no evidence of disease.

## 1. Introduction

Sarcomas comprise less than 2% of all primary salivary gland tumors [1]. Epithelioid hemangioendothelioma (EHE) is a rare tumor of endothelial differentiation occurring typically in adults with a wide age range and arising in many organs throughout the body, most notably in the liver, lungs, bones, and soft tissues. Histologically, it is characterized by epithelioid eosinophilic cells with cytoplasmic vacuolization (“blister cells”) arranged in cords or nests in a myxoid to hyaline stromal background [2]. Greater than 90% of cases demonstrate a t(1;3)(p36;q23-25) leading to *WWTR1-CAMTA1* fusion [3–5]. Only five cases of EHE arising in the salivary glands have been reported [6–10]. We report a primary EHE of the parotid gland presenting as ear pain and Bell's palsy, symptoms which have not been previously documented.

## 2. Case Report

A 37-year-old female was referred to otolaryngology by her primary care provider for chief complaint of right ear pain of two-year duration which reportedly began after a dog bite to the right lip and face. She had also experienced numbness in the right face from the lateral canthus to the jawline since the dog bite and reported that she had felt a mass in the right preauricular area for approximately five months. Six weeks prior to ENT consultation, she reported an episode of Bell's palsy, now with subjective incomplete recovery and residual facial asymmetry.

Physical exam revealed tenderness and fullness in the right preauricular region, but no definite mass, and weakness of the frontal and zygomatic/buccal branches of the right facial nerve, complete right eye closure, and near-normal symmetry at smile and cheek-blowing. Imaging was ordered at that time, but the patient did not follow up. Less than three weeks later, the patient complained of another episode of Bell's palsy, with an inability to close her right eye, followed by worsening symptoms including dry eye and blurry vision, right occipital headache, worsening right ear and facial pain, and increase in size of the preauricular fullness. Imaging was performed.

**Figure 1:**
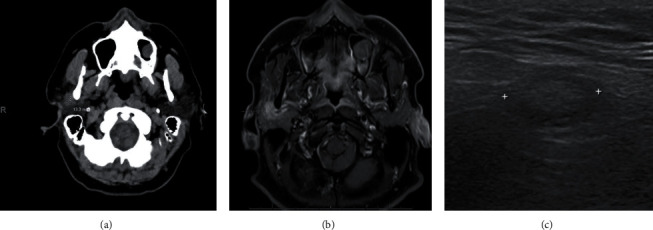
Imaging of the right parotid gland. (a) CT with contrast demonstrating increased soft tissue density. (b) MRI demonstrating mass-like enhancement. (c) Ultrasound demonstrating a poorly defined nodule.

CT with contrast revealed a focus of increased soft tissue density in the right parotid gland just lateral to the mandibular subcondylar region measuring 1.3 cm (Figure 1(a)). MRI showed an inflamed parotid gland with an area of enhancement possibly representing a mass. Inflammation and enlargement of the facial nerve was also noted (Figure 1(b)). Ultrasound showed an ill-defined hypoechoic nodule near the angle of the mandible/preauricular area (Figure 1(c)).

**Figure 2:**
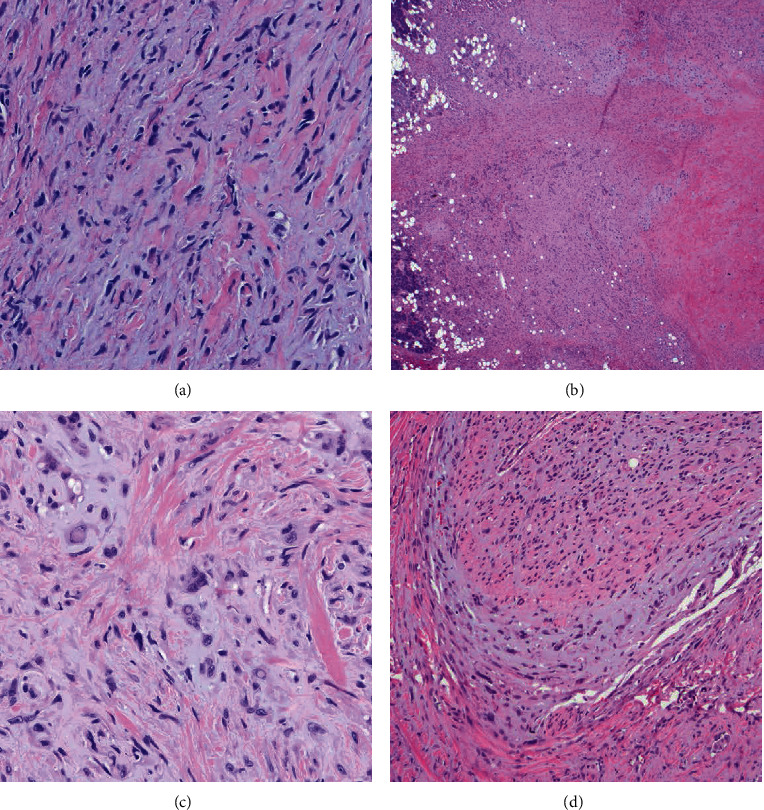
(a) Biopsy of the parotid gland revealed a neoplasm comprised of atypical cells in a fibromyxoid stroma (H&E^∗^, 400x). Resection of the parotid mass. (b) Tumor infiltrating adjacent tissue (H&E^∗^, 20x). (c) Tumor is comprised of atypical cells in small clusters with occasional intracytoplasmic vacuoles and foci of cytologic pleomorphism (H&E^∗^, 400x). (d) Perineural invasion (H&E^∗^, 100x). ^∗^Hematoxylin and eosin.

An ultrasound-guided fine needle aspiration of the right parotid gland was attempted, but the patient could not tolerate the procedure and there was insufficient material for diagnosis. Subsequent biopsy showed atypical cells embedded in a mostly myxoid background, highly suggestive of EHE (Figure 2(a)). However, this specimen was also very scant, and tissue was exhausted before a definitive diagnosis could be made.

Without a clear diagnosis, the patient underwent a right total parotidectomy and selective neck dissection. In the interval before surgery, she developed first bite syndrome and near complete paralysis of the superior division of the facial nerve. Intraoperatively, the superior division of the facial nerve grossly had been infiltrated with tumor and was sacrificed. The inferior division of the facial nerve was preserved. Due to technical constraints surrounding facial nerve preservation, the specimen was received as multiple unoriented fragments of tan-pink lobulated tissue. The largest fragment contained a 1.2 × 1.0 × 1.0 cm tan-white firm mass, free of the surgical resection margins.

**Figure 3:**
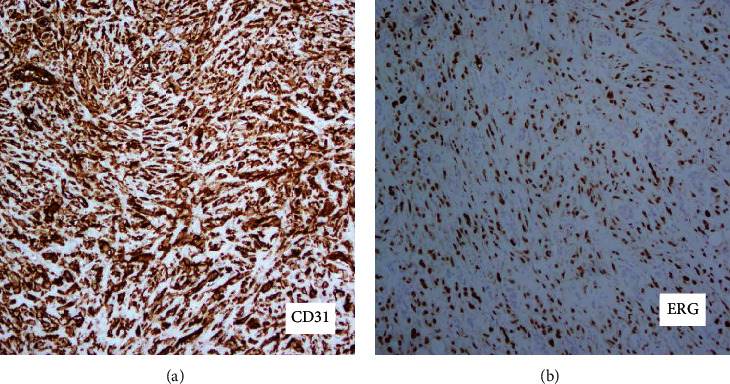
Positive immunohistochemistry for (a) CD31 and (b) ERG (100x).

Histologic sections demonstrated atypical single cells and occasional small clusters of cells in a fibromyxoid stroma. Some areas exhibited spindled features and foci of more marked cytologic atypia were also identified. Mitotic rate was less than 1/10 high-power fields. Multiple foci of perineural invasion were identified; however, lymphovascular invasion and tumor necrosis were not seen (Figures 2(b)–2(d)). Immunohistochemistry showed the tumor cells were positive for vascular markers ERG, CD31, and CD34 (Figures 3(a)–3(b)). Cytokeratin AE1/AE3 was negative.

Fluorescence in situ hybridization (FISH) of the tumor revealed a t(1;3)(p36;q25) involving the *CAMTA1* and *WWTR1* genes, confirming a diagnosis of epithelioid hemangioendothelioma. All 16 lymph nodes from the selective neck dissection were negative for tumor. No additional therapy was given. Follow-up PET imaging up to one year postsurgery demonstrated good response to surgical treatment in the right parotid bed with no evidence of residual/recurrent or metastatic tumor.

## 3. Discussion

Epithelioid hemangioendothelioma (EHE) is a rare mesenchymal tumor of endothelial differentiation [2, 11]. Over 90% of EHE harbor a t(1;3)(p36;q23-25) resulting in *WWTR1-CAMTA1* fusion [3–5]. A subset with distinct histologic features harbor a *YAP1-TFE3* translocation [12, 13]. EHE predominantly arises in deep somatic soft tissue, lung, and liver in adults, though it may arise in any tissue and has a wide age distribution. Though most follow a relatively indolent clinical course, overall about 20% will metastasize and 17% of patients will die of disease [2, 11, 14, 15]. Cases with the *YAP1-TFE3* translocation may represent a distinct clinical entity, with possibly more metastatic disease but an overall more indolent course [12, 13].

Mesenchymal tumors constitute 2-5% of all salivary gland tumors, and most are benign; approximately 0.3-1.5% of all salivary gland tumors are sarcomas. Most (about 80%) occur in the parotid gland [1]. Early comprehensive reviews of primary salivary gland sarcomas, including those seen at the AFIP and at the MD Anderson Cancer Center (from 1945 to 1985), both including literature reviews (approximately 120 cases in total), identified no cases of EHE of the salivary glands [16, 17]. In a subsequent literature review (1990 to 2010) and analysis of cases from MD Anderson Cancer Center (from 1990 to 2007), 187 primary sarcomas of the salivary gland were identified, including two cases of EHE from the literature (none from MD Anderson—these cases are discussed below) [1].

Only five cases of primary EHE arising in the salivary glands have been reported, all in the parotid gland [6–10]. It should be noted that in only one case was the diagnosis confirmed by assay for the *WWTR1-CAMTA1* (or *YAP1-TFE3*) translocation [8], though histologic features and immunohistochemical results were consistent with the diagnosis in all cases. Tumor size ranged from 1.5 to 4.2 cm. Only one patient demonstrated cranial nerve symptoms, exhibiting hypoglossal nerve paralysis on physical exam (specific details not provided). Perineural invasion was identified on pathologic examination in one additional case (Table 1).

Cranial nerve involvement (mainly facial nerve involvement) by salivary gland sarcomas does not seem exceptionally rare, outside of the general rarity of these sarcomas. In the 17 cases from the MD Anderson cohort of salivary gland sarcomas with clinical history, 2 presented with facial nerve symptoms, and 3 showed facial nerve invasion on histologic examination (though one of these was a rhabdoid neoplasm ex pleomorphic adenoma). Of those two primary sarcoma cases with facial nerve involvement, one patient died of disease within one year and the other was alive at 6.5 years [1]. By comparison, one review estimated the prevalence of facial nerve weakness or paralysis in malignant parotid gland tumors (almost all carcinomas) at 7-20% [18], though another demonstrated a prevalence up to 31% [19]. Of the six cases of primary EHE of the salivary gland (current case included), two exhibited cranial nerve symptoms, with one of these patients dying of disease (the only to do so in the series) (Table 1). It is unclear whether cranial nerve involvement in these cases did or did not contribute to the patients' poor outcomes.

Histologic features of EHE most highly associated with risk of metastasis and death from disease include size greater than 3 cm and/or mitoses greater than 3 per 50 HPF, with high risk tumors having a metastatic rate of 32% and 5-year disease specific survival of 59%. Fifteen percent of patients with low risk tumors developed metastatic disease, but none died [14]. Three cases of EHE of the salivary gland had one or both high risk features, including the only patient in this series with recurrent metastatic disease (to lung, liver, and spine at about five months after surgical treatment) who died of disease at 13 months [8]. The remaining patients were disease free at follow-up intervals ranging from 7 to 18 months (Table 1). Margins were specifically reported in only two cases (one negative and one positive, the latter in the patient with metastatic disease), and the remaining cases presumably had negative margins [6–10].

## 4. Conclusion

Compared to sarcomas of other sites, head and neck sarcomas have a higher local recurrence rate and worse disease-specific survival, possibly related to the difficulty of achieving wide resection margins in this region of delicate anatomy [20]. Based on their case series and review of the literature, Cockerill et al. concluded that local recurrence and distant metastases will develop in approximately 30-35% and 25-40%, respectively, in patients with primary salivary gland sarcomas [1]. How these data translate specifically to EHE in the salivary glands is unclear. As noted above, histologic features suggestive of more aggressive disease in EHE include size and mitotic activity. Traditional histologic features of aggressive behavior such as cytologic atypia and necrosis do not play a significant role in risk stratification as they do in other sarcomas, though the behavior of EHE can be difficult to completely predict. However, site of origin also plays an established role in prognosis in EHE. EHE arising in lung and bone is the most aggressive, while cutaneous tumors have a very good prognosis [2, 21–24].

There are too few salivary gland EHE cases to draw conclusions about site-specific prognosis. In this series, only one case behaved aggressively, which is broadly similar to the overall rate of aggressive behavior of EHE. While this case also had both high risk histologic features, two other cases demonstrating one or both features did not show recurrent or metastatic disease (though one case with both features did receive adjuvant radiation therapy as well as surgery, Table 1). Additional data are required to better understand the clinical behavior of EHE arising in salivary glands.

## Figures and Tables

**Table 1:** Characteristics of published cases of epithelioid hemangioendothelioma in the parotid gland.

	Current case	Suarez-Zamora et al. [9]	Koide et al. [8]	Falvo et al. [6]	Amin et al. [7]	Pigadas et al. [10]
Age, gender	37, female	62, female	70, female	28, male	81, male	48, female
Presenting signs/symptoms	24-month history otalgia, recent waxing-waning facial nerve palsy	12-month history slow-growing painless mass	18-month history swelling and pain	2-month history of rapidly enlarging lump with intermittent oppressive pain	6-month history initially asymptomatic mass; deep boring pain developed during workup	12-month history swelling with recent enlargement and intermittent sharp pain
Physical exam findings	CN VII paralysis and numbness; area of tenderness and fullness without definite mass	Mass partially fixed to neighboring tissues	Left CN XII paralysis; mass with overlying skin erythema & induration	Mass fixed to underlying planes	NR	Firm, semifixed, nontender mass
Size of tumor (cm)	1.2	1.8	4.2	3.8	2.0	1.5
Perineural invasion	+	NR	NR (presumed positive)	-	+	NR
Mitotic rate	<1 per 10 HPF	<3 per 50 HPF	5 per 50 HPF	5 per 10 HPF	>1 per HPF	“Inconspicuous”
Ki-67 (MIB-1) index	NR	5%	NR	6%	NR	“Negative staining”
Cytologic atypia	Marked	Absent	NR	Modest	Marked	Minimal
Necrosis	-	-	NR	-	+	NR
*WWTR1-CAMTA1* fusion	+	NR	+	NR	NR	NR
Margins	Negative	NR (presumed negative)	Positive	NR (presumed negative)	Negative	NR (presumed negative)
Treatment	Surgery (TP)	Surgery (SP)	Surgery (TP, node dissection), radiation	Surgery (TP, node dissection), radiation	Surgery (TP)	Surgery (SP)
Metastasis	No	No	None at presentation, died from distant mets seen at nearly 5 months in lung liver and lumbar spine	No	No	No
Outcome	NED 12 months	NED 12 months	DOD 13 months	NED 18 months	NED 7 months	NED 18 months

NR: not reported; HPF: high power field; TP: total parotidectomy; SP: superficial parotidectomy; NED: no evidence of disease; DOD: died of disease.

## Data Availability

All relevant data have been included in this manuscript.
